# The importance of truth: Joint retrieval of “true” and “important” feedback in multidimensional source memory

**DOI:** 10.3758/s13423-025-02702-9

**Published:** 2025-05-13

**Authors:** Daria Ford, Marek Nieznański

**Affiliations:** 1 Department of Psychology, School of Social Sciences, University of Mannheim, L13, 15, 68161 Mannheim, Germany; 2 Institute of Psychology, Cardinal Stefan Wyszyński University in Warsaw, Warsaw, Poland

**Keywords:** Source memory, Memory for truth and falsity, Value-directed remembering, Binding contextual information

## Abstract

Source memory for truth is usually better than for falsity, and similar effects are observed for important compared with unimportant information. A recently found interaction between information veracity and importance indicates that people effectively prioritize encoding true information (but not false). Yet it is unclear whether the feedback about veracity and importance of the information is integrated into joint memory representation. In the following experiment, we investigated whether source memory for veracity and importance dimensions is stochastically dependent. Students (*N* = 82) memorized trivia statements along with their veracity and importance status, which resulted in four different combinations of sources (“true and important”, “true and unimportant”, “false and important”, “false and unimportant”). The analysis with a multidimensional source memory multinomial model revealed that the joint retrieval of “true” and “important” feedback as compound information is better than for all other combinations. Moreover, the veracity dimension was memorized better than the importance dimension, showing that we remember whether information is true or false better than whether it is important or unimportant.

**Supplementary Information:**

The online version contains supplementary material available at 10.3758/s13423-025-02702-9.

Prioritization of incoming information plays a significant role in memorization: We remember important information better than unimportant (Cohen et al., 2017; Elliott & Brewer, 2019; Ford & Nieznański, 2024; Hennessee et al., 2017; Villaseñor et al., 2021; Yin et al., 2021), and this effect is usually referred to as "the effect of value” (e.g., Yin et al., 2021). Moreover, the source (context) of the presentation of the important information is usually also remembered better than contextual details of unimportant information (e.g., Cohen et al., 2017; Ford & Nieznański, 2024, Yin et al., 2021). The source defined in these memory studies can be colored images in the background of words, font color of the presented words (Yin et al., 2021), the plurality status (word presented in singular or plural form; Cohen et al., 2017), but also the feedback accompanying the statement—whether a statement was important or unimportant (Ford & Nieznański, 2024).

In research on memory for truth and falsity, source can be determined by feedback indicating whether the statement was true or false (e.g., Niedziałkowska & Nieznański, 2021). Participants are usually presented with trivia statements (e.g., “The biggest aggregation of desert salt is in Iran.”) along with the “true” or “false” feedback and later asked during the test phase to recognize if the statement was presented and what feedback was provided during the study phase (Ford & Nieznański, 2023, 2024; Nadarevic & Erdfelder, 2013, 2019; Niedziałkowska & Nieznański, 2021). Interestingly, memory for the truth of the information is usually better than for the falsity (Ford & Nieznański, 2023, 2024; Nadarevic & Erdfelder, 2019, Experiment 2; Niedziałkowska & Nieznański, 2021; but see Nadarevic & Erdfelder, 2013; Nadarevic & Erdfelder, 2019, Experiment 1), and we will refer to this effect as “the effect of truth” (Ford & Nieznański, 2024).

Our previous findings have suggested that one reason we remember truth better than falsity may be an automatic tendency to perceive true information as important (Ford & Nieznański, 2023, 2024). The following article aims to answer whether we create a joint memory representation for feedback information that a statement is true and important, which results in stochastic dependency between “true” and “important” information retrieval.

## Why is truth important by default?

We have recently compared “the effect of value” and “the effect of truth” and found that they similarly affect underlying memory processes (Ford & Nieznański, 2024). Moreover, we found that people can effectively prioritize memorizing “true” feedback information but not “false” feedback information. The probable mechanism underlying the observed enhanced memory for valuable information is more attentional resources being allocated to the prioritized information (Allen, 2019). The same is valid for true information: When attentional refreshing was suppressed, the effect of truth was diminished, meaning there was no advantage of context memory for truth over falsity (Ford & Nieznański, 2023). This result provides a piece of evidence that attentional refreshing is indeed involved in the effect of truth. Recently, Yin et al. (2024) found that another attention maintenance mechanism—namely, articulatory rehearsal—is involved in the effect of value. However, when articulatory rehearsal was suppressed while processing true and false statements, the difference in memory performance was only numerical, providing no evidence that articulatory rehearsal enhances context memory for true information (Ford & Nieznański, 2023).

A recent study by Nadarevic (2025) compared memory for true and false statements presented as either definitions (e.g., “The mastodon is an extinct proboscidean”) or comparatives (e.g., “The carbon dioxide emissions per capita are higher in Finland than in Estonia”). When statements were framed as definitions, people tended to remember “true” feedback better than “false.” However, this effect was diminished in the case of comparatives, likely because even false comparative statements can still convey useful information. This finding implies that the effect of truth may be a specific instance of a broader effect of relevance since higher memory performance seems to occur for statements that are relevant, not just true. A similar perspective is provided by the devaluation-by-inhibition hypothesis proposed by Raymond et al. (2005), which describes how distractors are evaluated more negatively (devalued) compared with targets. According to the authors, this occurs because the selection of targets requires active inhibition of distractors. In line with the devaluation-by-inhibition hypothesis, Santos et al. (2017) demonstrated that information presented with the forgetting cue in the item-method directed forgetting paradigm was judged as less true. This suggests that false information may be actively devalued and, as a result, cannot be prioritized at encoding. Additionally, Mayo et al. (2004) showed that false tags may work as an external negation, evoking inhibitory processes. Therefore, false or unimportant information may be actively inhibited, while true or important information receives greater attention/additional rehearsal during encoding, contributing to the observed memory performance patterns.

In sum, we can expect that only true and important information is relevant, and that such information may be selectively rehearsed or, alternatively, irrelevant information may be inhibited at encoding. Both ways of processing lead to enhanced memory only for important and true sentences. Since “false and important” or “true and unimportant” information are irrelevant to our goals, we are not motivated to rehearse them or we actively inhibit (devaluate) them.

## The present study

This experiment aimed to conceptually replicate and expand the previous findings of Ford and Nieznański (2024) while using a different theoretical framework. Previously, we manipulated prioritization between subjects by assigning participants to one of two groups where either truth or falsity was prioritized. All participants encountered true and false statements. In the current design, we manipulated *veracity* and *importance* at the same time, crossing two levels of value with two levels of veracity feedback. This allows us to make more justified conclusions about the relationship between the effects of value and truth. Moreover, we employed a multidimensional source memory multinomial model (Meiser, 2014), which is an extension of the two-high-threshold model by Bayen et al. (1996). We expected to find an increased joint retrieval for “true and important” information compared with any other combination of sources (“true and unimportant”, “false and important”, “false and unimportant”). We predicted that “true and important” sources would be remembered better than “true and unimportant” sources following the previous results on the effect of value, where the context memory for important (more valuable items) was better than for unimportant ones (Ford & Nieznański, 2024; Yin et al., 2021). Based on the results from our earlier study (Ford & Nieznański, 2024), we predicted that people would not be able or motivated to prioritize false information. Moreover, if only “true and important” information is treated as relevant and automatically bound, we predicted that the conflict between “false and important” or “true and unimportant” sources may arise. “False and unimportant” sources should have acquired the least attention (rehearsal) or strongest inhibition while encoding, which would result in the worst joint memory retrieval.

## Methods

### Participants

A group of 82 undergraduate students (20 men, *M*_age_ = 20 years, *SD* = 1.72, range: 18–29) participated in the study. All of them were given partial course credits for their participation. The experiment was programmed in E-Prime 2.0 software (Psychology Software Tools, Pittsburgh, PA, USA) and run in the university lab. Five participants, at the same time, at separated individual stations, completed the study, which took around 10–12 min. We accepted all students from the course willing to participate in the study to allow them to earn optional course credits. All participants provided informed consent before the experiment started. We employed a convenient sample of psychology students and collected as many participants as possible from the same cohort.

### Materials

Materials were prepared based on trivia statements taken from previous studies (e.g., Corneille et al., 2020; Nadarevic et al., 2018), and we also generated some new statements. Our goal was to select two versions of each statement, both moderately plausible: one true (e.g., “Children have more bones than adults”) and one false (e.g., “Adults have more bones than children”) to account for any possible differences between them. The materials and the detailed description of the selection procedure can be accessed at the OSF repository: https://osf.io/b8zte (materials); https://osf.io/nq5d9 (description). For the experiment, we selected the top-scoring pairs of statements for the study phase. In the test phase, as distractors, we added 16 top-scoring individual statements not included in any pair.^1^

### Procedure

In the study phase, participants were presented with 36 statements along with the information about *veracity* (true/false) and *importance* (important/unimportant). This resulted in four different types of sources: “true and important”, “false and important”, “true and unimportant”, and “false and unimportant”. In the assignment of two dimensions, our goal was to not create any discrepancies between them. Therefore, while validating material, we chose it to be independent of previous knowledge (hence, moderate plausibility) and individual’s priority preference (consequently, we assigned the importance a priori).^2^ Three buffer statements were shown at the beginning and another three at the end of the study phase. Statements were presented sequentially for 7 s in white font on a black background, centered in Times New Roman size 32, with an interstimulus interval of 250 ms. We counterbalanced the material by creating eight different versions based on: the order of the source presentation (true/false and important/unimportant or important/unimportant and true/false), veracity status (true or false), and importance status (important or unimportant).

Before the test phase, participants were explained, as in the research by Meiser and Bröder (2002), the meaning of “Remember” and “Know” answers. The instructions were translated into Polish and can be accessed at the OSF repository (https://osf.io/j4e9k). In the test phase, participants were presented with a set of 54 statements, of which 18 were new. In the first step of the memory test, participants had to indicate whether the item was old or new. In the case of a “new” answer, they proceeded with the next test item. If the answer was “old,” participants were asked to indicate whether they recollected it (“Remember”) or not (“Know”). In the last step of the memory test, participants were asked to indicate the source; one of four combinations possible on two dimensions: *veracity* (true or false) and *importance* (important or unimportant).

During data analysis, two pairs of statements appeared to have been presented with the wrong veracity value. Considering that the material was pretested, and statements of moderate plausibility were selected, we decided not to exclude them from the analyses.

### Multidimensional model of source memory (Meiser, 2014)

Multinomial processing tree (MPT) models were developed to differentiate between item and source memory (e.g., Batchelder & Riefer, 1999; Bayen et al., 1996; Riefer et al., 1994). They allow disentangling various latent memory processes from response guessing (for reviews, see Batchelder & Riefer, 1999; Erdfelder et al., 2009; for a tutorial, see Schmidt et al., 2023).

**Fig. 1:**
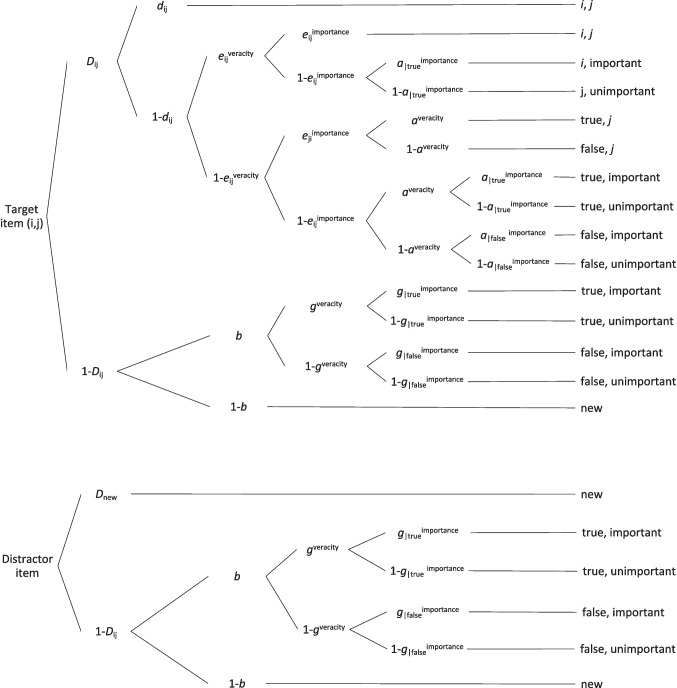
Multinomial processing tree diagram of the multidimensional source memory model. *Note*. This figure is adapted from “Analyzing Stochastic Dependence of Cognitive Processes in Multidimensional Source Recognition,” by T. Meiser, 2014, *Experimental Psychology, 61*(5), p. 408, where *D*_ij_ = probability of correctly recognizing target items from the sources *i* (*veracity*: true/false) and* j* (*importance*: important/unimportant); *d*_ij_ = probability of a correct joint retrieval of source combination *i*, *j* of a recognized item (“true and important”, “true and unimportant”, “false and important”, “false and unimportant”); *e*_ij_^veracity^ = probability of independent retrieval of source (true, false) on the *veracity* dimension; *e*_ij_^importance^ = probability of independent retrieval of source (important, unimportant) on the *importance* dimension; *a*^veracity^ = probability of guessing “true” on the *veracity* dimension for recognized target items; *a*_|true_^importance^, *a*_|false_^importance^ = probability of guessing “important” on the *importance* dimension for recognized target items assigned to true or false, respectively; *g*^veracity^ = probability of guessing “true” on the *veracity* dimension for unrecognized target or distractor items; *g*_|true_^importance^, *g*_|false_^importance^ = probability of guessing “important” on the *importance* dimension for unrecognized target or distractor items assigned to true or false, respectively; *b* = probability of guessing “old”; *D*_new_ = probability of recognizing distractor items as new

The multidimensional MPT model of source memory allows for measuring more than one source dimension and testing whether features from different dimensions are bound as compound information. Such a binding is reflected in the stochastic-dependent retrieval. This occurs when the retrieval of one contextual feature is more probable if the other contextual feature is retrieved successfully (Arnold et al., 2019; Meiser, 2014; Meiser & Bröder, 2002). In the current experiment, this model allowed for the measuring and comparing of joint retrieval in two dimensions, *veracity*: true and false, and *importance*: important and unimportant. Employing the multidimensional source memory model (Meiser, 2014) allowed verification of our main hypothesis, as it estimated the probability of joint retrieval of *veracity* and *importance* dimensions. The model and a detailed description of the parameters of the model are presented in Fig. 1.

## Results

The primary focus of our research is the multinomial model parameters that represent the joint retrieval of both source dimensions’ features. The data and code are available at the OSF repository (https://osf.io/ucs34) and (https://osf.io/pq3wj), raw data; (https://osf.io/txe4r), code. In this study, we followed hierarchical Bayesian MPT modeling, which accounts for the heterogeneity of the individual participants’ results (Heck et al., 2018). As recently argued by Arnold et al. (2019), such an approach rules out the possibility that stochastic dependence of source dimensions retrieval stems from a spurious correlation due to aggregation of data across participants.^3^ We adopted the latent-trait approach (Klauer, 2010), which uses the multivariate normal distribution of the transformed individual parameters as the prior distribution on a group level. The parameter posterior estimates were obtained using Markov chain Monte Carlo sampling methods (Plummer, 2003). Analyses were conducted using the R package TreeBUGS (Heck et al., 2018) with 50,000 iterations.^4^ Stochastic dependence in source information retrieval is specifically observed in the state of recollection (Meiser, 2014; Meiser et al., 2008; Meiser & Bröder, 2002), so we focused on “Remember” responses analysis. Parameter estimates for both “Remember” responses and combined “Remember” and “Know” responses can be found in Table A in the Appendix. Before assessing model fit, we imposed several constraints on parameters. Such restrictions have been used in many previous applications of this model (e.g., Arnold et al., 2019). We equated detection parameters: *D*_new_ = *D*_true,important_; guessing parameters: *g*^veracity^ = *a*^veracity^, *g*_|true_^importance^ = *a*_|true_^importance^, *g*_|false_^importance^ = *a*_|false_^importance^; and independent source retrieval parameters,^5^ which under these constraints transformed into memory for source dimensions *veracity* (*e*^veracity^) and *importance* (*e*^importance^): *e*_true,__important_^veracity^ = *e*_false,__important_^veracity^ = *e*_true,__unimportant_^veracity^ = *e*_false,__unimportant_^veracity^*, e*_true,__important_^importance^ = *e*_false,__important_^importance^ = *e*_true,__unimportant_^importance^ = *e*_false,__unimportant_^importance^.

**Fig. 2:**
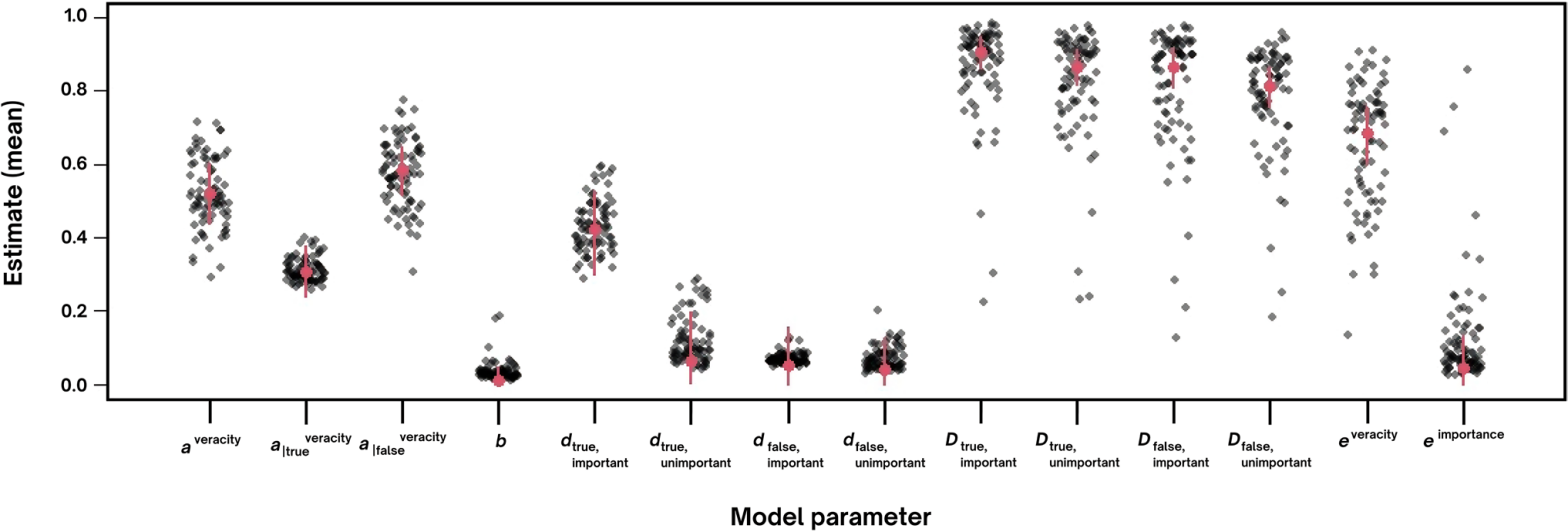
Group-level means + 95% BCI (red) and individual means (gray). (Color figure online)

Model fit was assessed with posterior-predictive *p* values and indicated a satisfactory fit of the model predictions to both observed mean frequencies (*T*_*1*_: *p* = .557) and the covariance structure across participants (*T*_*2*_: *p* = .209). The parameter estimates and the corresponding Bayesian credibility intervals (BCIs) of the MPT model for multidimensional source information are presented in Fig. 2.

### Hypotheses testing

#### Memory for joint sources

The 95% BCIs for parameter estimates cannot include zero; however, the lower bound was very close to zero for estimates for *d*_true, unimportant_, 95% BCI = [.002, .20], *d*_false, important_, 95% BCI = [.001, .16], and *d*_false, unimportant_ , 95% BCI = [.001, .13]. In contrast, the 95% BCI for estimate of *d*_true, important_ was [.30, .52], This clearly shows that joint retrieval for sources other than “true and important” was minimal or nonexistent. The descriptive pattern based on median scores for joint source memory, indicated a difference between “true and important” combination and all other combinations: *Median d*_true,important_ = .42, *Median d*_true, unimportant_ = .06, *Median d*_false, important_ = .05, *Median d*_false, unimportant_ = .04. In other words, the participants showed memory for “true and important” source, whereas other combinations were memorized vaguely or not at all. To test for differences in parameters between joint retrieval of “true and important” source compared with other configurations of source features we sampled the parameter differences, Δ*d* = *d*_true,important_ − *d*_true, unimportant_, Δ*d1* = *d*_true,important_ − *d*_false, important_, Δ*d2* = *d*_true,important_ − *d*_false, unimportant_. If the 95% BCI for the parameter difference estimate does not contain zero, there is evidence for a difference. For Δ*d* = .35, 95% BCI = [.15, .49], for Δ*d1* = .37, 95% BCI = [.20, .49], and for Δ*d2* = .38, 95% BCI = [.22, .50], showing a substantial difference between joint retrieval of source “true and important” and all other sources (“true and unimportant”, “false and important”, “false and unimportant”). This test showed that the “true and important” source was memorized better than any other combination of sources.

### Exploratory analyses

#### Memory for dimensions: Veracity and importance

As an exploratory analysis, we tested for differences in parameters between independent retrieval of source dimensions *veracity* and *importance*. We sampled the parameter difference, Δ*e* = *e*^veracity^ – *e*^importance^. For Δ*e* = .64, 95% BCI = [.53, .73], indicating a substantial difference. This means that the *veracity* dimension was generally remembered better than the *importance* dimension.

#### Memory for statements

We also tested statement memory but failed to find any substantial differences between the statement types: Δ*D* = *D*_true,important_ − *D*_true, unimportant_ = .04, 95% BCI = [− .01, .10], Δ*D1* = *D*_true,important_ − *D*_false, important_ = .04, 95% BCI = [− .02, .10], Δ*D2* = *D*_true,important_ − *D*_false, unimportant_ = .05, 95% BCI = [− .01, .11]. This means that we found no differences in memory performance between the statement types.

## Discussion

The study applied the multidimensional source memory model to measure the joint retrieval of *veracity* and *importance* for the first time. The results show better memory for joint retrieval of “true and important” sources than any other combinations. This suggests that “true and important” combination of features on the dimensions of *veracity* and *importance* creates a special type of binding, leading to improved retrieval. This finding supports our previous claims that indicating something is true is equivalent to assigning it a higher value, while indicating something is false is like assigning it a lower value (Ford & Nieznański, 2024).

The enhanced joint retrieval of “true and important” feedback demonstrates that prioritizing truth increases its relevance. However, this effect does not apply in the case of inconsistent feedback, such as when falsity is prioritized or truth is unimportant. In such cases, we assign low or even null relevance to information that is either unimportant or false.

The lack of the effect of truth or the effect of value for inconsistent combinations of feedback may be due to various basic cognitive mechanisms. For example, only information that is both true and important may be automatically assimilated into knowledge, as it is most valuable. Participants may also strategically allocate resources by selectively rehearsing only information that is both true and important, as falsity tagged as important or truth tagged as unimportant is not worth investing cognitive resources in. Recent findings by Yin et al. (2024) have highlighted the role of not only attentional refreshing, but also articulatory rehearsal in encoding important information. Similar effects have been found for the enhanced recollection of true (compared with false) information (Ford & Nieznański, 2023).

Another possibility is that forgetting of configurations with “false” or “unimportant” feedback is due to inhibition, similar to what has been postulated for items with a “forget” cue in the item-method directed forgetting paradigm (e.g., Jing et al., 2019). This way, “false” feedback or “unimportant” feedback may trigger an active inhibitory mechanism regardless of whether the feedback is “true” or “important.” Directed forgetting processes have mostly been studied for item memory; however, some research has also suggested that a “forget” cue can also induce source forgetting (Thompson et al., 2011; see Hourihan, 2021). Consistent with our findings, Abel and Bäuml (2023, Experiment 3) have recently demonstrated that intentional forgetting of headlines reduces memory regardless of whether these headlines are marked as trustworthy or untrustworthy. Santos et al. (2017) have shown that to-be-forgotten statements are perceived as less true, indicating devaluation in the truth judgments. The authors highlighted the adaptiveness of the inhibition processes in assigning relevance to the information.

Our results also show increased memory for the dimension *veracity* compared with *importance*. This indicates memory for the veracity of information as a relevant source dimension for people to memorize information. This possibly aligns with the observation that comprehension and validation are a unified process (Richter, 2015; Van Moort et al., 2020; but see Wiswede et al., 2013). This means that while processing the information, people judge its plausibility to decide whether it is worth encoding. Another possibility is that *veracity* draws more attentional resources because this feature is an internal feature of an item and cannot be easily changed; true statements will (rather) never become false ones. In a philosophical context, veracity is typically defined as the logical value of an item. This corresponds with the idea of Grover (1992) who argued that a statement indicating something is true functions only as a form of drawing attention to another statement that is rich in content, as the truth statement does not provide any content itself. The truth statement (feedback, in our research) is rather a linguistic tool to enhance the meaning of another statement associated with it, so it is more about the structure and economy of language usage. Importance, on the other hand, can be easily changed depending on the goal of encoding and individual interpretation. In this study, importance was defined a priori, on a group rather than individual level, so the manipulation of importance might not have been so strong as it was not related to the participant’s personal goals or values.

We did not find differences in memory for the statements (item memory), so we did not confirm earlier findings on better memory for important statements compared with unimportant ones (Ford & Nieznański, 2024; Hennessee et al., 2017; Villaseñor et al., 2021; Yin et al., 2021). This could be due to participants being near ceiling-level performance for statement recognition. Another reason for that can be related to the presentation context and possibly weaker *importance* manipulation compared with *veracity* manipulation.

There has been criticism in the literature concerning the idea of direct binding of two sources (Starns & Hicks, 2005, 2008). It has been proposed that sources are bound to the item instead of to each other directly (Nieznański et al., 2024; Starns & Hicks, 2008). The purpose of this study was not to address the controversy over the direct or indirect nature of source information binding. However, we did demonstrate a clear piece of evidence for stochastic dependence in the retrieval of “true and important” information. It seems unlikely that it was due to a factor affecting source and item memory simultaneously since item memory was not enhanced for the “true and important” configuration. On the other hand, the other parameters representing joint retrieval of sources were near zero, suggesting that for other combinations of source dimensions, a model containing solely independent source memory parameters would be sufficient.

In summary, our results show for the first time that “true” feedback is effectively bound with “important” feedback information, resulting in joint retrieval of source information. This confirms claims from our earlier research (Ford & Nieznański, 2024) based on a different paradigm that truth and importance are easily translated as highly relevant contexts. This study supports the finding that people can effectively prioritize memorizing “true” feedback information but not “false” feedback information. In line with this, we find that binding of “true” feedback information with “unimportant” cue or “false” feedback with “important” cue is completely ineffective. The mechanism of eliminating the effect of value on source memory for false information and reducing the effect of truth for unimportant information requires further investigation, which, for example, would consider the role of inhibitory processes and selective attentional maintenance.

## Supplementary Information

Below is the link to the electronic supplementary material.

Supplementary file1 (DOCX 19.3 KB)

## Data Availability

The raw data and materials are available at the Open Science Framework (https://osf.io/ucs34) and (https://osf.io/pq3wj), raw data; (https://osf.io/b8zte), materials.

## Footnotes

1 This solution was not optimal, as noted by the reviewer, and the assignment of old versus new statements should have been counterbalanced.

2 As noted by the reviewers, this choice of assignment might not have been ideal, as veracity is a more objective dimension, whereas importance is generally more impactful when personalized. However, manipulating personal importance would be technically difficult and could introduce confounding factors, such as the self-reference effect (Nieznański, 2009; Symons & Johnson, 1997).

3 Nevertheless, we decided to conduct MPT analyses on aggregated data and a post hoc power analysis. We were interested in the robustness check of our results and also the sensitivity analysis, which indicated the obtained power. These analyses were conducted in multiTree (Moshagen, 2010) and can be found on the OSF repository (https://osf.io/q5jhz).

4 The other parameters were left as default (e.g., 2,000 burn-in iterations and three chains).

5 Sensitivity analysis on aggregated data showed that such a reduction in *e* parameters was necessary. With all *e* parameters set free, to achieve a power of 1 − β = .80 to detect Δ = .19 difference between *d* parameters, we would need to gather observations from about 1,800 participants.

## Code availability

The code is available at the Open Science Framework (https://osf.io/txe4r).

## Declarations

### Open practice statement

The data, analysis code, and materials for the experiment are available at the Open Science Framework (https://osf.io/tf9ah/).
