# Supplementary material for: The importance of truth: Joint retrieval of “true” and “important” feedback in multidimensional source memory
Source: Psychon Bull Rev. 2025 May 13;32(5):2344–52. doi: 10.3758/s13423-025-02702-9 (PMC12426068; doi:10.3758/s13423-025-02702-9)
Supplement: Supplementary file 1 — Supplementary file1 (DOCX 19.3 KB) [file 13423_2025_2702_MOESM1_ESM.docx]

**Appendix**

**Table A**

*Group-level medians parameter estimates with standard deviations and 95% Bayesian Credibility Intervals of the multidimensional source memory model*

| Parameter | Remember | Remember and Know |
| --- | --- | --- |
| *D*_true, important_ | .91 (.02) [.86, .95] | .91 (.02) [.88, .94] |
| *D*_true, unimportant_ | .87 (.03) [.81, .91] | .88 (.02) [.83, .92] |
| *D*_false, important_ | .87 (.03) [.81, .92] | .88 (.02) [.83, .92] |
| *D*_false, unimportant_ | .81 (.03) [.76, .87] | .84 (.02) [.79, .89] |
| *d*_true, important_ | .42 (.06) [.30, .52] | .35 (.05) [.24, .43] |
| *d*_true, unimportant_ | .06 (.05) [.00, .20] | .07 (.05) [.00, .19] |
| *d*_false, important_ | .05 (.04) [.00, .16] | .05 (.04) [.00, .14] |
| *d*_false, unimportant_ | .04 (.03) [.00, .13] | .03 (.03) [.00, .10] |
| *e*^veracity^ | .68 (.04) [.60, .76] | .58 (.04) [.50, .65] |
| *e*^importance^ | .04 (.04) [.00, .13] | .07 (.04) [.01, .15] |
| a^veracity^ | .52 (.04) [.44, .60] | .52 (.04) [.46, .59] |
| a_\|true_^importance^ | .31 (.04) [.24, .38] | .28 (.03) [.22, .34] |
| a_\|false_^importance^ | .58 (.03) [.52, .65] | .52 (.03) [.46, .57] |
| b | .10 (.01) [.00, .05] | .09 (.04) [.02, .19] |
| Model fit: Mean structure (*T1*) | p = .557 | p = .458 |
| Model fit: Covariance structure (*T2*) | p = .209 | p = .190 |

*Note*: Values are group-level medians with standard deviations in parentheses and 95% Bayesian Credibility Intervals (BCIs) in brackets. Parameters of the multidimensional source memory model were obtained using TreeBUGS (Heck et al., 2018). “Remember and Know” responses were modeled jointly.
